# Analyzing the Donor Dilemma: Outcomes of Kidney Transplant Recipients From Donors With Positive Blood Cultures Obtained at Organ Procurement

**DOI:** 10.1093/ofid/ofae694

**Published:** 2025-02-27

**Authors:** Petros A Svoronos, R Alfonso Hernandez Acosta, Prakhar Vijayvargiya, Pradeep Vaitla, James Wynn, Christopher Anderson, Jason Parham, Elena Beam, M Rizwan Sohail, Zerelda Esquer Garrigos

**Affiliations:** Division of Infectious Diseases, University of Mississippi Medical Center, Jackson, Mississippi, USA; Division of Infectious Diseases, Department of Medicine, Massachusetts General Hospital, Boston, Massachusetts, USA, Harvard Medical School; Division of Infectious Diseases, University of Mississippi Medical Center, Jackson, Mississippi, USA; Division of Nephrology, Department of Medicine, University of Mississippi Medical Center, Jackson, Mississippi, USA; Division of Transplant and Hepatobiliary Surgery, Department of Surgery, University of Mississippi Medical Center, Jackson, Mississippi, USA; Division of Transplant and Hepatobiliary Surgery, Department of Surgery, University of Mississippi Medical Center, Jackson, Mississippi, USA; Division of Infectious Diseases, University of Mississippi Medical Center, Jackson, Mississippi, USA; Division of Infectious Diseases, Mayo Clinic College of Medicine and Science, Rochester, Minnesota, USA; Division of Infectious Diseases, Mayo Clinic College of Medicine and Science, Rochester, Minnesota, USA; Division of Infectious Diseases, Baylor College of Medicine, Houston, Texas, USA; Division of Infectious Diseases, University of Mississippi Medical Center, Jackson, Mississippi, USA; Division of Infectious Diseases, Mayo Clinic College of Medicine and Science, Rochester, Minnesota, USA

**Keywords:** bacteremia, donor, infection, prophylaxis, transmission

## Abstract

Based on expert consensus, the American Society of Transplantation recommends 7–14 days of preventive antibiotic therapy for solid organ transplant recipients from donors with positive blood cultures. We evaluated management and outcomes of kidney transplant recipients from these donors.

Despite the benefits of transplantation for survival and quality of life, infections remain a major cause of disease and death. These infections often result from surgical complications, nosocomial exposure, or transmission from the donor [1]. Although the incidence of donor-derived bacterial infections in kidney transplant recipients (KTRs) is relatively low [2], mortality rates can reach 10% [3], especially in cases involving multidrug-resistant (MDR) organisms [4]. However, a previous study suggested that solid organs from donors with positive blood cultures can be safely transplanted if recipients receive prophylactic antimicrobial therapy after transplantation [5].

Organ Procurement and Transplantation Network policies require a variety of screening tests for transmissible disease in all deceased donors around the time of organ procurement, which includes blood cultures. Guidelines from the American Society of Transplantation (AST) recommend administering 7–14 days of preventive antibiotic therapy (PAT) to transplant recipients who receive organs from bacteremic donors [6]. However, this recommendation is primarily based on expert consensus, as there are no published studies addressing the necessity and optimal duration of PAT in this context. While PAT may reduce the risk of infectious complications, it carries potential adverse effects, including the development of antibiotic resistance. Organ Procurement and Transplantation Network policies require a variety of screening tests for transmissible disease in all deceased donors around the time of organ procurement, which includes blood cultures. The current study examined management and outcomes in KTRs from donors with positive surveillance blood cultures obtained at the time of organ procurement.

## METHODS

A single-center, retrospective study was conducted from 1 January 2015 to 31 December 2020, including all KTRs aged ≥18 years who received organs from donors with positive surveillance blood cultures at the time of organ procurement. Donor data were confirmed and extracted from UNet.

For the purpose of this study, “bacteremia” was defined as the presence of typically pathogenic bacterial species identified in ≥1 blood specimen obtained via culture [7]. Common commensal organisms were classified in accordance with the Centers for Disease Control and Prevention's National Healthcare Safety Network's list of organisms [8]. MDR organisms were defined as any isolate with documented resistance to ≥1 agent in ≥3 separate antimicrobial categories [9]. Donor blood cultures with growth of fungal organisms were excluded from the study, as were donors with “true,” clinically apparent bacteremia identified before organ procurement. The primary outcome was the rate of infection within 30 days of transplantation caused by the same organism identified in the donor's surveillance blood cultures.

The study was approved by the University of Mississippi’s institutional review board (no. 2020V0403). Written consent from participants was not required.

## RESULTS

Of the 675 KTRs during the study period, 56 received kidneys from donors who had positive surveillance blood cultures. The median age at transplantation (interquartile range [IQR]) was 58 (36.8–79.2) years, and sex distribution was equal between male and female KTS (28 of 56 [50%] for both). The majority of KTRs were black (47 of 56 [83.9%]).

The organisms identified in donor surveillance blood cultures are listed in Table 1. Of the 56 identified cases, 36 (64.3%) were categorized as common commensals, while the remaining 20 (35.7%) were classified as true bacteremia. Six donors had polymicrobial growth.

**Table 1.** Organism Isolates and Outcomes of Preventive Antibiotic Therapy

Donor Blood Culture Isolate	KTRs, No. (%)^a^		
	KTRs (n = 56)	KTRs With PAT (n = 23)	KTRs Without PAT (n = 33)
Gram-positive organisms isolated (commensals excluded)	11	9	2
Gram-negative organisms isolated	12	8	4
***Donor cultures with non-commensal organisms***	20 (35.7)	14 (60.9)	6 (18.2)
**Organisms isolated**^b^	26	19	7
* Acinetobacter baumannii* complex	2 (7.6)	0	2 (28.6)
* Chromobacterium violaceum*	1 (3.8)	1 (5.3)	0
* Clostridium sordellii*	2 (7.6)	2 (10.6)	0
* Enterobacter cloacae* complex	1 (3.8)	0	1 (14.3)
* Enterococcus faecalis*	1 (3.8)	0	1 (14.3)
* Escherichia coli*	2 (7.6)	2 (10.6)	0
* Fusobacterium nucelatum*	2 (7.6)	2 (10.6)	0
* Klebsiella pneumoniae*	2 (7.6)	2 (10.6)	0
* *Microaerophilic streptococci	1 (3.8)	0	1 (14.3)
* Pseudomonas aeruginosa*	1 (3.8)	0	1 (14.3)
* Staphylococcus aureus*	6 (23.7)	5 (26.3)	1 (14.3)
* Streptococcus pneumoniae*	4 (15.4)	4 (21)	0
* *Probable *Veillonella* species	1 (3.8)	1 (5.3)	0
*** *Outcomes**			
Positive cultures within 30 d^c^	4 (20)	2 (14.3)	2 (33.3)
Positive urine cultures within 30 d	3 (15)	2 (14.3)	1 (16.7)
Bacteremia within 30 d	0	0	0
Same organism as donor cultures^c^	0	0	0
MDR organism after transplantation	0	0	…
Duration of PAT, median (IQR), d	7 (0–7)	8.5 (7–14)	…
Organism potentially susceptible to PAT^d^	12 (46)	12 (85.7)	…
Organism potentially susceptible to perioperative antibiotics^d^	12 (46)	12 (85.7)	1 (16.7)
Organism potentially susceptible to TMP/SMX^d^	9 (34.6)	9 (64.2)	1 (16.7)
***Donor cultures with commensal* *organisms*** ^e^	36 (64.3)	9	27
**Organisms isolated**^b^	39	12	27
Coagulase-negative *Staphylococci*	26 (66.6)	4 (44.4)	22 (81.5)
*Streptococcus anginosus* group	3 (7.7)	3 (33.3)	0
*Actinomyces* species	2 (5)	2 (22.2)	0
*Cutibacterium* species	2 (5)	0	2 (7.4)
*Dermacoccus nishinomiyaensis*	2 (5)	0	2 (7.4)
Viridans group streptococci	4 (10.3)	3 (33.3)	1 (3.7)
**Outcomes**			
Positive cultures within 30 d^c^	11 (30.6)	2 (22.2)	9 (33.3)
Positive urine cultures within 30 d	7 (19.4)	1 (11.1)	6 (22.2)
Bacteremia within 30 d	3 (8.3)	0	3 (11.1)
Same organism as donor in cultures^c^	0	0	0
MDR organism after transplantation	0	0	0
Duration of PAT, median (IQR)	0 (0–0.25)	7 (5–14)	…
Organism potentially susceptible to PAT^d^	9 (25)	9 (100)	…

Abbreviations: IQR, interquartile range; KTRs, kidney transplant recipients; MDR, multidrug resistant; PAT, preventive antibiotic therapy.

^a^Data represent no. (%) of KTRs unless otherwise specified.

^b^Two donors had polymicrobial culture growth with mixed commensal and noncommensal organisms, 1 had multiple noncommensal organisms and 1 had multiple commensal organisms.

^c^Including blood, urine, sputum, wound, and fluid cultures.

^d^Susceptibility based on confirmed donor culture susceptibilities or potentially covered based on typical susceptibilities.

^e^Common skin commensal as listed by the Centers for Disease Control and Prevention’s National Healthcare Safety Network.

All KTRs received a single dose of perioperative antibiotics at the time of transplantation, mostly cefazolin (44 of 56 [78.6%]). After transplantation, most patients were prescribed trimethoprim-sulfamethoxazole (TMP/SMX) prophylaxis for *Pneumocystis jirovecii* (51 of 56 [91%]) at the institution's standard dose of 80 mg/400 mg, adjusted for creatinine clearance.

### KTRs From Donors With True Bacteremia

Of the 20 KTRs from donors with true bacteremia, 14 (75%) received PAT for a median (IQR) duration of 8.5 (7–14) days. The organisms identified in the 6 cases where PAT was not administered included *Acinetobacter baumannii*, *Enterobacter cloacae*, *Enterococcus faecalis*, *Pseudomonas aeruginosa*, and *Staphylococcus aureus*.

Notably, donor culture susceptibilities were not available for all patients who received PAT, and only 7 (50%) received therapy tailored to specific susceptibility results. However, in 12 cases (85.7%), the prescribed PAT was likely effective against the organism isolated from the donor blood cultures based on typical susceptibility profiles. In addition, perioperative antibiotics would have covered the organism in 12 cases (85.7%), while TMP/SMX prophylaxis would have provided coverage in 9 (64.2%). Among the 6 cases in which PAT was not prescribed, only 1 recipient whose donor cultures grew *S aureus* would have been potentially covered by perioperative antibiotics and TMP/SMX prophylaxis.

### KTRs From Donors With Common Commensals

In contrast to cases with true bacteremia, most KTRs from donors with positive surveillance blood cultures for common commensal organisms (27 of 36 [75%]) were not treated. Among the 9 who did receive PAT, the median (IQR) duration of antibiotic therapy was 7 ( 5–14) days. Of these, only 5 (55.6%) received therapy based on available susceptibility data. However, all 9 received coverage that was expected to be effective against the organisms based on typical susceptibility profiles.

### Route of Antibiotic Administration

Among the 23 KTRs who received PAT, 17 were treated with oral antibiotics, including 10 who received a combination of oral and intravenous antibiotics (Table 2). For KTRs with commensal organisms, the majority (7 of 9 [77.8%]) received treatment that included an intravenous antibiotic. Similarly, among KTRs with bacteremia, 10 of 14 (71.4%) received treatment that included an intravenous antibiotic.

**Table 2.** Antibiotic Used for Preventive Antibiotic Therapy

PAT Antibiotic	KTRs, No. (%) (n = 23)^a^	Median PAT Duration, d
Intravenous^a^	18	
Vancomycin	7 (38.9)	7
Penicillin G	1 (5.6)	7
Ampicillin	1 (5.6)	3
Cefazolin	3 (16.7)	7
Ceftriaxone	3 (16.7)	14
Ceftazidime	1 (5.6)	14
Cefepime	2 (11.1)	14
Oral^a^	17	…
Linezolid	2 (11.8)	14
Clindamycin	1 (5.9)	7
TMP/SMX	1 (5.9)	7
Minocycline	1 (5.9)	7
Levofloxacin	5 (29.4)	10
Metronidazole	4 (23.5)	12
Amoxicillin	2 (11.8)	10.5
Cephalexin	1 (5.9)	7

Abbreviations: KTRs, kidney transplant recipients; PAT, preventive antibiotic therapy; TMP/SMX, trimethoprim-sulfamethoxazole.

^a^Ten KTRs received combination intravenous and oral PAT.

### Outcomes

No infections with the same isolate as identified in donor surveillance blood cultures were reported within the first 30 days after transplantation in either group. However, infections with a different organism were observed in 4 cases (25%) among the 20 with bacteremia and in 11 cases (30.6%) among the 36 with common commensal organisms. In addition, no infections with MDR organisms were identified.

## DISCUSSION

While the AST offers guidance regarding treatment of recipients of donors with positive blood cultures, these recommendations are largely based on expert opinion rather than robust data from clinical trials. The lack of high-quality research addressing the indications for PAT—specifically optimal duration and route of administration—complicates clinical decision making in this population. These uncertainties can result in either undertreatment or overtreatment, both of which pose significant risks to patients.

In our study, no infection transmission was observed among KTRs from donors with positive surveillance blood cultures, despite receipt of PAT at the lower end of the AST's recommended duration. Furthermore, 26% of KTRs (6 of 23) were treated exclusively with oral antibiotic regimens (Table 2). This suggests that, in select cases, oral antimicrobials may be sufficient for preventing transmission and could potentially obviate the need for intravenous catheter placement for outpatient therapy.

In addition, most KTRs whose donor cultures grew commensal organisms did not receive PAT, and, similarly, no infections were documented in this cohort. This suggests that for donors with positive surveillance blood cultures yielding commensals, treatment may not be needed given the low risk of transmission. However, it is important to recognize that some commensal organisms can exhibit pathogenic potential in certain cases. Thus, understanding the clinical context of the donor is crucial when determining whether treatment of the recipient is warranted.

Interestingly, in 6 recipients from bacteremic donors who did not receive PAT, infection did not develop, despite their donors having growth of virulent pathogens, such as *S aureus* and *P. aeruginosa*. Of these cases, only 1 recipient whose donor cultures grew *S aureus* would have been potentially covered by perioperative antibiotics and TMP/SMX prophylaxis. Although these limited data do not allow for definitive conclusions, it suggests that the transmission risk of pathogenic organisms may be lower than previously assumed. This observation aligns with another study wherein 29 recipients (5%) received organs from donors with bacteremia that was recognized only after transplantation, with no evidence of infection transmission [10]. However, due to the small patient sample, further studies are needed to refine current practices. Moreover, it remains uncertain whether the low doses of TMP/SMX administered to prevent *P. jirovecii* pneumonia are sufficient to effectively treat or prevent bacterial infections.

The current study has several limitations. Its retrospective design and small sample size limit the generalizability of the findings. In addition, antibiotic susceptibility data from donor blood cultures were unavailable in most cases, as was information on antibiotic treatment that they received. Furthermore, information regarding whether the treating clinicians had access to donor cultures results or the factors guiding their clinical decision making was not provided. Individual characteristics of recipients that could also influence outcomes, such as immunosuppressive therapy, were beyond the scope of our study.

Finally, as previously noted, our study exclusively includes KTRs from donors with positive surveillance blood cultures obtained around the time organ procurement, with the exact timing in relation to transplantation unavailable. This distinction is important, as these cultures are typically obtained as part of routine screening rather than in response to clinical symptoms of infection in the donor. Consequently, the findings of our study may not apply to cases in which donors had positive blood cultures before screening or “true,” clinically apparent bacteremia, which would warrant a different and individualized approach.

In conclusion, in KTRs whose donors had positive surveillance blood cultures at the time of organ procurement, there was no transmission of infection up to 30 days after transplantation despite most receiving PAT at the lower end of the AST's recommended duration. Moreover, the majority of KTRs from donors with common commensal organisms were not treated and also had no transmission of infection. In select cases, oral antimicrobials may suffice to prevent transmission and avoid the need for intravenous antibiotic therapy. Larger, multicenter studies are required to confirm these findings and optimize treatment guidelines.
